# Effects of plant community structural characteristics on carbon sequestration in urban green spaces

**DOI:** 10.1038/s41598-024-57789-2

**Published:** 2024-03-28

**Authors:** Xuguang Zhang, Hengshuo Huang, Ke Tu, Rui Li, Xinyu Zhang, Peng Wang, Yonghua Li, Qiusheng Yang, Aidan C. Acerman, Nan Guo, Yang Liu

**Affiliations:** 1 College of Landscape Architecture and Art, Henan Agricultural University, Zhengzhou, China; 2 College of Environmental Science and Forestry, State University of New York, Syracuse, NY USA; 3 International Education College, Henan Agricultural University, Zhengzhou, China; 4 Zhengzhou-China Greening Expo Management Center, Zhengzhou, China

**Keywords:** Density, Coverage, Structural characteristics, Optimal regulation, Forestry, Urban ecology, Environmental impact

## Abstract

The structural characteristics of plant communities in urban green spaces have a significant impact on their carbon sequestration function. In this study, comprehensive data were collected from 106 plant communities (each 20 m × 20 m) in Zhengzhou Green Expo Park. We assessed aboveground and soil carbon storage, alongside maintenance carbon emissions, to quantify carbon dynamics. Our primary objective was to establish a statistical model that correlates the structural attributes of plant communities with their total annual carbon sequestration. This model aims to provide a quantitative framework for optimizing community structures to maximize carbon sequestration in urban green spaces. The results showed that density and coverage were significantly and positively correlated with aboveground and soil carbon stocks. Density and mean height were significantly and positively correlated with maintenance carbon emissions. Density played a key structural role in regulating the total carbon sequestration of the plant communities, being 27.24 times more effective than coverage. The total annual carbon sequestration of the plant community reached an optimal value of 327.67 kg CO_2_-eq/y^−1^ at a density and cover of 0.15 and 1, respectively. This study provides valuable data for increasing the carbon sink ability of urban green spaces through plant structure regulation and supporting low-carbon development strategies in urban management.

## Introduction

Green spaces play a vital role as natural carbon sinks, significantly contributing to the reduction of atmospheric CO_2_ levels and mitigating the impacts of climate change on urban environments^1^. The efficiency of carbon sequestration in urban green spaces' plant communities is influenced by three key factors: direct atmospheric carbon absorption through plant photosynthesis. Photosynthesis is the process where plants absorb sunlight through chlorophyll in their chloroplasts and convert water and carbon dioxide into sugars and oxygen. In this process, sunlight is absorbed by chlorophyll, used to break down water molecules, and releases oxygen into the atmosphere. At the same time, plants absorb carbon dioxide from the air, which combines with the broken-down water to form sugars and other organic materials, providing energy for plant growth; biomass carbon accumulation during plant growth; and the soil’s decomposition and storage capabilities for atmospheric carbon. Li^2^ found that the annual carbon storage of existing vegetation leaves was 4.24 million tons in the green space of 8 built-up urban districts in Beijing. The study by Zhang in 16 urban district green spaces in Shanghai showed that the annual biomass carbon storage was 1.44 million tons^3^. Guan et al.’s study in Guangzhou showed that the soil carbon storage in urban green areas in Guangzhou was 12.32 million tons^4^.

The structural combination characteristics of the plant community impact its carbon storage ability. Nowak et al. concluded that community density is proportional to the greenfield carbon sequestration benefit, among other influential factors, but too high a density reduces the space for individual tree growth, which increases tree mortality and decreases the carbon sequestration benefit^5^. Stoffberg et al. suggested that the carbon sequestration benefit of vegetation is related to the diameter at breast height (DBH, and when vegetation is at a young age, the amount of carbon sequestration is not that high; however, the growth of vegetation represents an increase in biomass; therefore, the vegetation carbon sequestration efficiency depends on the growth rate^6^. Lautenbach et al.^7^ showed that plant community species richness had a linear relationship with carbon sequestration, and the green space coverage could be increased based on the relationship between carbon sequestration and species richness. In addition, habitat and microclimate changes influenced by planting structure differences, such as light, wind, temperature and humidity conditions, also impact community carbon storage functions^5^. Sagar et al. concluded that the plant carbon sequestration capacity is related to forest canopy closure extent, light intensity, and soil moisture based on measurements of basic plant and environmental indicators in tropical forests in India^9^. Yu et al. found that planting density affected the intensity of light received by plants in the plant community, and the higher the plant density, the stronger the carbon sequestration capacity of the urban green space^10^. Zhu et al. found that the carbon sequestration of plant communities with different planting densities in Harbin's urban green spaces differed significantly, and the carbon sequestration of plant communities with high planting densities was significantly higher than that of plant communities with low planting densities^11^.

Carbon emissions from urban green spaces mainly come from planting construction and maintenance, where planting construction is a one-time carbon emission and the latter accounts for continuous carbon emissions. Maintenance work generates carbon emissions through water and fertilizer application or fossil fuel consumption of maintenance equipment (chainsaws, hedge trimmers, trucks). The removed vegetation is eventually decomposed by the soil. Part of the carbon in plants is released to the atmosphere and part is stored in the soil^12^. Jo and McPherson^13^ estimated carbon sequestration in green spaces in residential areas in Chicago as 26.15 kg/m^2^/y and concluded that urban tree planting has a positive impact on reducing the atmospheric carbon content. McPherson^14^ quantified the carbon benefits of an urban forest in Sacramento, California, which offset 0.29 t/ha of carbon emissions per year, and proposed a management strategy for urban forests. By analysing soils in urban green spaces, Dib et al. concluded that soil carbon storage can help to mitigate the continued increase in anthropogenic carbon emissions and reduce the climate change potential^15^. Although vegetation and soil can absorb carbon from the atmosphere, the intensive management of urban green spaces is usually accompanied by higher carbon emissions, therefore, optimization strategies should be proposed for urban green space management in terms of energy conservation and emission reduction^5^.

Current research on carbon sequestration in urban green spaces has predominantly focused on comprehensive carbon accounting across various urban scales. For example, the average annual carbon sequestration of urban green space in the United States, which stands at 22.8 million tons Mt, urban forest carbon sequestration in Shanghai, recorded at 2.87 Mt^3,14^ and the total annual carbon sequestration of 6 parks and green areas in Zhengzhou was reported as 294,684 t^16^. However, there is still a lack of clarity in exploring the specific relationship between the structural attributes of plant communities and their carbon storage capacity. This study aims to address this research gap by focusing on 106 plant communities in Zhengzhou Green Expo Park. Our research hypothesis posits that changes in the structural characteristics of these plant communities significantly affect their carbon sequestration capacity. To test this hypothesis, we will quantify the annual amounts of aboveground and soil carbon storage, as well as maintenance carbon emissions. We seek to establish a clear statistical relationship between modifications in community structure characteristics and variations in carbon sequestration. In addition, we aim to propose a quantitative structural adjustment method to improve the carbon storage capacity of plant communities in urban green spaces.

## Materials and methods

**Figure 1:**
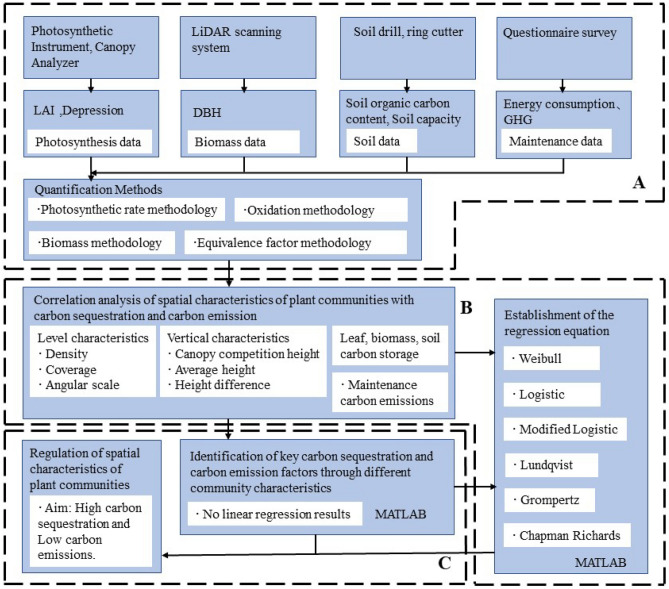
Technical workflow.

The main technical processes in this paper include. A: data collection of above-ground and soil carbon storage and maintenance carbon emissions from community sample squares and quantification methods for carbon sequestration. B: Data analysis using MATLAB_R2021a (https://www.mathworks.com/products/matlab.html). C: Optimal structural characteristic regulation strategies for increasing the carbon sequestration capacity at the plant community scale of urban green spaces (Fig. 1).

### Research area and sample establishment

**Figure 2:**
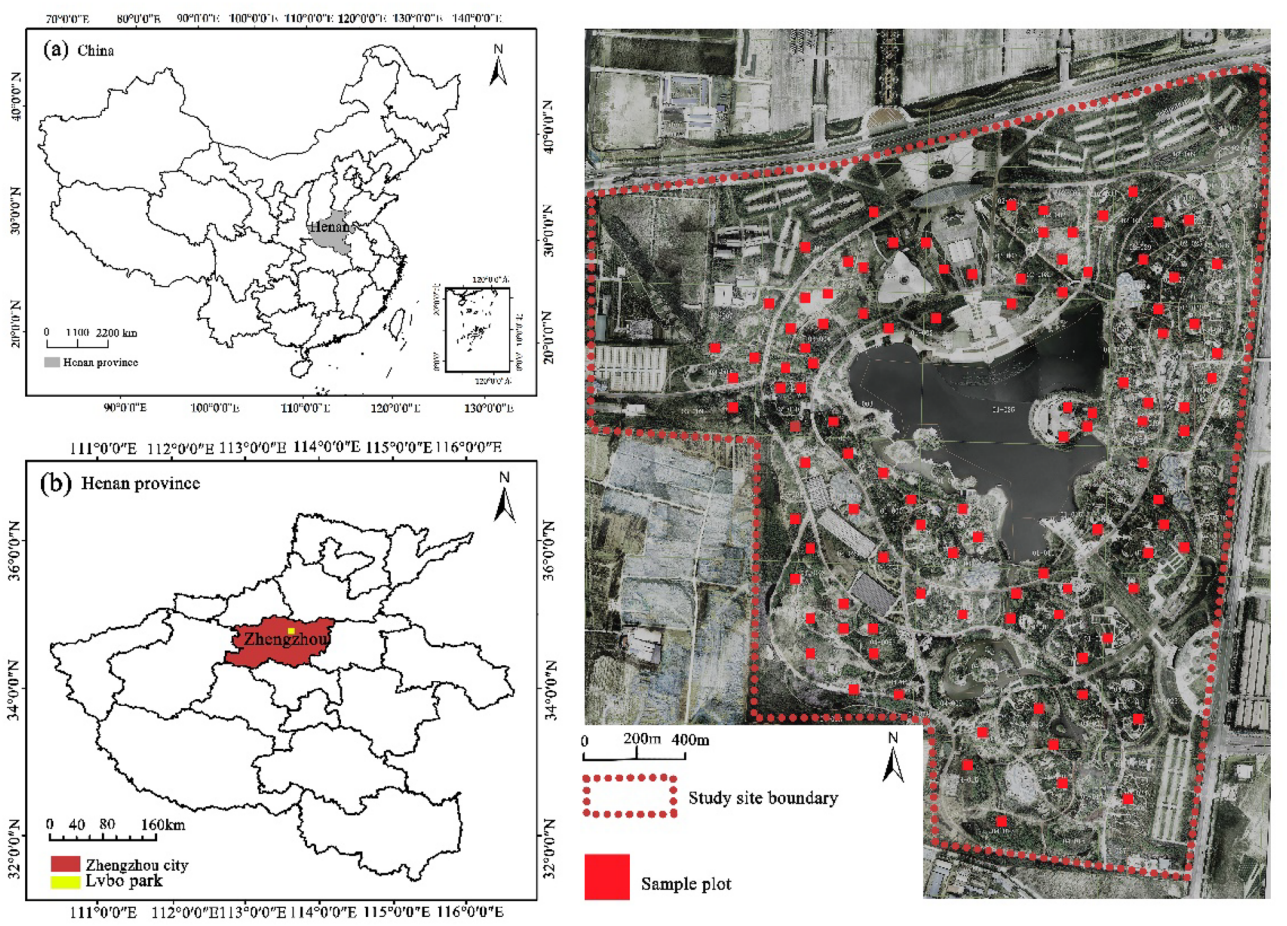
Study area and the location of the plant community sample plots. Note: Figure for ArcMap10.2 software production (https://www.arcgis.com/index.html).

The research area is located in Zhengzhou City, Henan Province (112°42′–114°13′E, 34°16′–34°58′N) within Green Expo Park, with a typical warm-temperate continental monsoon climate, an average annual temperature of 14.4 °C, an average annual precipitation of 640.9 mm, a frost-free period of 220 days, and about 2400 h of sunshine throughout the year. The total area of the study area totals 196 ha. The planting work of the park was completed in 2010 and is a mature green space. Green Expo Park is rich in water resources, the east side of the base is adjacent to the Yellow Diversion Canal, the southwest and the Jialu River near; the park terrain is flat, high in the southwest, low in the northeast, the difference in elevation is less than 2 m, the soil is mainly sandy soil, the culverted water layer is between 4–6 m. A total of 106 plant communities with different spatial structures were randomly selected in the study area (Fig. 2). The community composition of the samples was dominated by trees and shrubs, with stable growth and located in terrestrial areas (This study was conducted with the permission of Zhengzhou-China Greening Expo Management Center and Henan Agricultural University).

**Figure 3:**
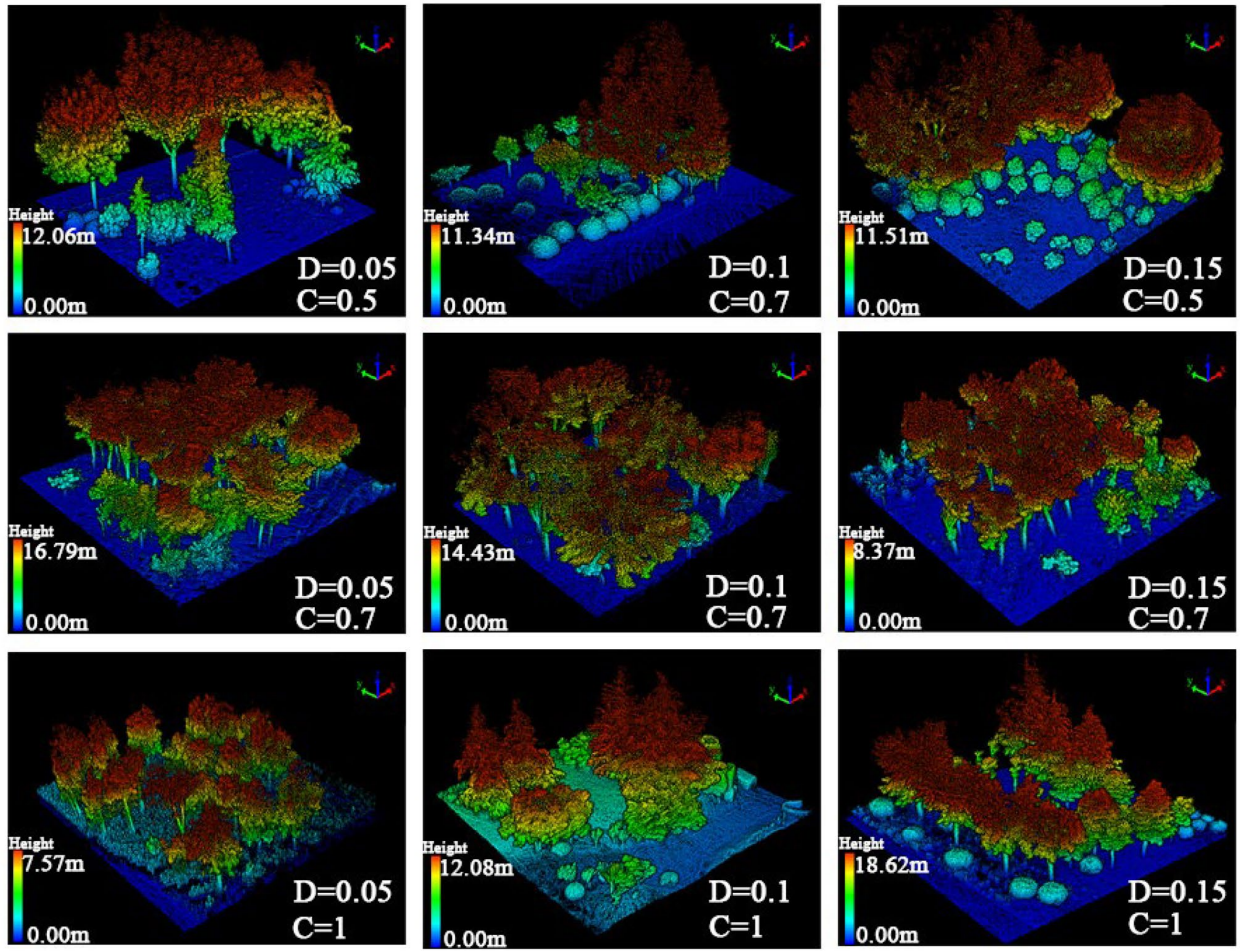
Sample LiDAR models with different structural characteristics. (D represents density; C represents coverage. This map point cloud sample is consistent with the regulation strategy map below).

The LiBackpack system was used to collect point cloud data of the Zhengzhou Green Expo park and separate the 106 plant communities from the research area using LiDAR360 point cloud operation software. LiBackpack used a top-down scanning method by setting up LiDAR sensors in the horizontal and vertical directions and was equipped with high-precision GNSS equipment, combined with simultaneous localization and mapping to build SLAM (simultaneous localization and mapping) technology to obtain high-precision 3D point cloud data within the scanning area (Fig. 3)^17^.

### Site experimental data acquisition

A sunny, windless, and naturally well-lit day was selected for the leaf photosynthetic rate experiment, aimed at qualification of the photosynthetic carbon storage in the plant community. Three mature leaves of each plant that were robust, of similar growth, and on the sunlight side were selected for measurement. The photosynthetic rate of each leaf was determined based on 5–10 instantaneous measurements taken each time and repeated three times every hour from 9:00 a.m. to 18:00 p.m. using a photosynthesis meter. After three days of measurements per plant, the average photosynthetic rate of individual plants in the community was calculated^18^ (this article states that the plant material used in the study complies with relevant agency, state and national guidelines and regulations).

A total of 106 soil samples were collected in order to be able to clearly analyze the differences in soil carbon stocks of different plant communities. The surface of each on-site sample was cleared of mulch for surface soil collection and prepared for the soil carbon experiment. The soil was sampled from the bottom up along the diagonal direction of each grid using a 5 cm diameter soil auger, and a total of 5 samples were taken from each soil layer, i.e., 0–20 cm, 20–40 cm^19^. The five samples from the same soil layer were mixed well and combined into one soil sample (the aim was to average the variability of soil carbon stocks across soil depths), passed through 2 mm sieve to remove plant residues, gravel, and other debris, and air-dried in a greenhouse to measure the soil physicochemical indexes^20^.

Questionnaire research and field measurements were used to collect maintenance task and workload data about plant maintenance to calculate the maintenance carbon emissions for the green space samples (in administering the questionnaire, we ensured that informed consent was obtained from all participants). We collected a total of 106 questionnaires from all the samples. The collected maintenance data mainly included annual workload data (2022.02-2022.10) for pruning, irrigation, fertilization, pesticide, and litter removal. Every maintenance task included the investigation of equipment application, the operation time in different seasons, the material type and input amount, and an exhaust emission composition test. (All methods were carried out in accordance with the relevant guidelines. All experimental protocols were approved by Zhengzhou-China Greening Expo Management Center and Henan Agricultural University. This statement is made with the consent of all authors of the paper and Henan Agricultural University).

### Data processing

The focus of this paper is on the relationship between plant community structure factors as a function of community carbon storage, so when making the selection of plant structure factors, it is necessary to describe the three-dimensional structural characteristics of plant communities in detail. First, the plant communities in urban green spaces were selected according to the horizontal structure (density, coverage, and angular scale). The reason is that the different structural characteristics of the plant community and its various ecological functions also have greater differences. Selecting density, coverage, and angular scale as the structural factors of the plant community can describe the number of individuals, the area covered, and the distribution pattern of the plant community, and analyze the functional relationship between them and the carbon storage. Secondly, the plant communities in urban green space were selected according to vertical structure (average height of trees, height difference of community, and canopy competition height), because it was found that the maintenance workload of plant communities with multi-layer structure was higher than that of single-layer structure, and the difficulty of maintenance work of high-level plant communities was higher than that of bottom-layer plant communities; however, the carbon storage of multi-layer structure and high-level plant communities was generally higher than that of single-layer and bottom-layer plant communities. Therefore, the selection of the average height of the trees, the height difference of the community, and the height of the competition of the canopy can describe the vertical structure characteristics of the plant community in detail.

### Quantification of the structural characteristics of the plant community

Comparative shortest-path (CSP) algorithm to extract CSV tables of the morphological structure parameters of single woody plants, including the tree height, DBH, crown area (CA) and crown diameter (CD) values. The CSV tables were able to directly generate the density, coverage, mean height and height difference characteristics of the sample plant community^21–23^.

Angular scale reflects the distribution pattern of tree species within the plant community and their arrangement in horizontal position, it is a parameter describing the spatial structure, which determines the distribution pattern of tree species by describing the uniformity of neighboring wood around the reference tree, and analyzes the distribution of forest trees by comparing the angle of intersection constituted by the reference tree and its neighboring wood (α with the expected angle of entrainment in the case of a uniform distribution (the standard angle α = 72°)). $w$ is calculated as

1 $$\begin{array}{*{20}c} {w = \frac{1}{N}\mathop \sum \limits_{i}^{N} w_{i} } \\ \end{array}$$

*w* in the formula denotes the angular scale. where N is 4 and represents the four directions around the object woody plant. Wi is the distribution indicator, calculated by the Penman equation^24^. When w_i_ = 0 and w_i_ = 0.25 the plant community is uniformly distributed; when w_i_ = 0.5, it is randomly distributed; and when w_i_ = 0.75 and w_i_ = 1, it is unevenly distributed.

Canopy competition height (CCH) is the height above which the leaves of a tree can fully utilize the intensity of solar radiation above them for photosynthesis. The principle is to categorize each tree in a plant community into different height levels by the differences in height and crown width of individual plants. The CCH was calculated as^25^:

2 $$\begin{array}{*{20}c} {CCH = \alpha L + Hw} \\ \end{array}$$

where a is the cut-off coefficient (0.6), L is the crown diameter, and *Hw* represents the height beneath the branch of a single woody plant, measured from the plant community LiDAR model.

#### The aboveground carbon storage quantification of the plant community

With reference to previous studies, the aboveground carbon storage measurements for trees and shrubs were divided into the leaf photosynthetic function and body cumulative biomass^26^.

The CO_2_ and moisture contents of the leaves were measured to obtain the net assimilation per unit leaf area in a certain time period, the photosynthetic CO_2_ fixation per unit land area was obtained based on the leaf area index (LAI), and the sample total photosynthetic CO_2_ storage was simulated based on the depression of the plant community. In this study, mid-autumn time was chosen for photosynthetic data measurements because it is representative of the time of year when it is closest to the year's average temperature (15 °C). Photosynthesis data measurements lasted three days, and the average carbon storage of plants during these 3 days was obtained to represent the daily carbon storage in the plant growth season as follows^27^ As shown in Appendix A (Table A1):

3 $$\begin{array}{*{20}c} {p = \frac{i}{\it \Sigma }\left[ {\left( {p_{i + 1} + P_{i} } \right)/2 \times \left( {t_{i + 1} - t_{i} } \right) \times 3600/1000} \right]} \\ \end{array}$$

where p is the daily CO_2_ assimilation per unit area, in mmol/m^2^; P_i_ and p_i+1_ represent the instantaneous photosynthetic rate at the initial and next measurement points, respectively, in µmol/m^2^/s. t_i_ and t_i+1_ represent the time at the initial and next measurement points, respectively, on an hourly basis.

4 $$\begin{array}{*{20}c} {w_{{{\textit{CO}}_{2} }} = p \times \left( {1{-}20\% } \right) \times 44/1000} \\ \end{array}$$

${\text{w}}_{{{\text{CO}}_{{2}} }}$ represents the mass of CO_2_ stored per unit area of leaves, in g/m^2^-d, and 0.044 is the molar mass of CO_2_.

5 $$\begin{array}{*{20}c} {W_{d} = w_{{{\text{CO}}_{2} }} \times L \times CA} \\ \end{array}$$

${\text{W}}_{{\text{d}}}$ is the daily photosynthetic CO_2_ storage of a single plant, in g/d-1. L is the plant LAI, and CA represents its canopy area in m^2^.

6 $$\begin{array}{*{20}c} {LCS_{d} = \frac{i}{\Sigma }\left( {W_{di} \times C_{i} } \right)/C} \\ \end{array}$$

LCS_d_ is the daily photosynthetic carbon storage in the plant community, in g/d-1 CO_2_-eq. C_i_ is the depression of the ith plant, and C is the total depression of all plants in the community^28^.

According to the meteorological data of Zhengzhou city, the annual LCS in this paper was calculated only for sunny weather; rainy days were not included^29^.

The dry weight of the existing plants was measured based on the biomass model to indirectly calculate the plant biomass carbon storage^26^. Since the research object of this paper was the tree and shrub communities, a suitable biomass model was selected according to the plant DBH class. We used four biomass models to calculate trunk biomass, branch biomass, and root biomass, and leaf biomass was quantified using the photosynthetic rate method. As shown in Appendix A (Tables A2, A3):

7 $$\begin{array}{*{20}c} {B = 0.8 \times \Sigma B_{i} /S} \\ \end{array}$$

where B is the community biomass, in kg/hm^2^; S is the sample area, in hm^2^; B_i_ is the total biomass of the plant, and 0.8 represents the adjustment factor. As biomass is calculated as the dry weight of plants, which contain a high amount of organic matter in addition to water and a small amount of minerals, and their elemental composition is dominated by carbon, hydrogen and oxygen, researchers generally adopt 0.5 as the average carbon content (${\upalpha }$); therefore^30^.

8 $$\begin{array}{*{20}c} {BCS = \alpha \times B} \\ \end{array}$$

where BCS is the carbon storage in kg/hm^2^ CO_2_-eq. The plant anisotropic growth equation was applied in this paper to calculate the average annual biomass difference in the plant community to generate the annual BCS of the research samples were obtained in Appendix A (Tables A1–A3).

#### The soil carbon storage quantification of the plant community

9 $$\begin{array}{*{20}c} {SCS = \frac{SOC \times BD \times D}{{10}}} \\ \end{array}$$

where SCS is soil carbon storage, in mg/hm^2^ CO_2_-eq; SOC is the soil organic carbon content, in g/kg, which was determined by the K_2_Cr_2_O_7_-H_2_SO_4_ oxidation method from the soil samples. BD is the soil bulk weight, Soil bulk weight shall be called dry bulk weight, also known as soil pseudo-specific gravity, which refers to the ratio of the mass of a certain volume of soil (including the soil particles and the pore space between the particles) after drying to the volume before drying. in g/cm^3^; and D is the soil thickness of the research samples, in cm. This study referenced the value difference of the same date in mid-October by a one-year cycle to represent the annual SCS in the sample plant community.

#### The maintenance carbon emissions quantification of the plant community

Carbon emissions from plant communities mainly includes the carbon emissions of maintenance material input and equipment operation. The numerical calculation of the energy consumption (${\text{A}}_{{{\text{w}}i}}$) of maintenance equipment powered by petrol and diesel is as follows^31^:

10 $$\begin{array}{*{20}c} {A_{wi} = \mathop \sum \limits_{a}^{n} \frac{{M_{wa} }}{{E_{ea} }} \times \beta_{a} } \\ \end{array}$$

where a is the maintenance equipment associated with the input material *i* in the maintenance tasks. ${\text{M}}_{{{\text{w}}a}}$ refers to the annual workload of maintenance equipment $a$, and ${\text{E}}_{{{\text{e}}a}}$ and $\beta_{a}$ represent the working efficiency of equipment a and the amount of energy consumed per time unit, respectively. Table 1 shows the efficiency and carbon emission inventories of the equipment used in the field maintenance phase of the green space sample, and the data were obtained from field experiments^32,33^.

**Table 1:** Working efficiency and carbon emissions of the major maintenance equipment.

Equipment type	Energy consumption type	Work efficiency	Energy consumption (kg/h)	GHG emission (kg/h)		
				CO_2_	CH_4_	N_2_O
Power chain saw	Petrol	100 m^2^/h	0.80	1.55	9.28 × 10^−3^	5.46 × 10^−4^
Hedgerows	Petrol	300 m^2^/h	0.60	1.69	8.96 × 10^−3^	3.20 × 10^−4^
3 m^3^ loading light truck	Diesel	20 km/h	1.00	3.43	8.11 × 10^−3^	5.22 × 10^−4^
5 m^3^ loading truck	Diesel	20 km/h	2.60	6.20	1.81 × 10^−3^	9.39 × 10^−4^

The annual maintenance carbon emission of the plant community in the urban green space (${\text{MCE}}_{{\text{y}}}$, in kg CO_2_-e) was calculated as

11 $$\begin{array}{*{20}c} {MCE_{y} = \mathop \sum \limits_{i}^{n} \alpha_{ci} \times A_{wi} + EI_{eg} } \\ \end{array}$$

where $\alpha_{ci}$ is the carbon emission potential equivalent coefficient of the *i*th maintenance material and ${\text{EI}}_{{{\text{eg}}}}$ is the carbon emission quantity of the maintenance equipment used in the plant community, calculated as follows:

12 $$\begin{array}{*{20}c} {EI_{eg} = \mathop \sum \limits_{a}^{n} \frac{{M_{wa} }}{{E_{ea} }} \times \left( {A\gamma_{a} \times A\alpha_{ci} + B\gamma_{a} \times B\alpha_{ci} + C\gamma_{a} \times C\alpha_{ci} } \right)} \\ \end{array}$$

where ${\text{A}}\gamma_{a}$, ${\text{B}}\gamma_{a}$, and ${\text{C}}\gamma_{a}$ represent the carbon emission factors CO_2_, CH_4_, and N_2_O, respectively, from the operation of the maintenance equipment. ${\text{A}}\alpha_{ci}$, ${\text{B}}\alpha_{ci}$, and ${\text{C}}\alpha_{ci}$ represent the equivalent coefficients of the carbon emission factors CO_2_, CH_4_, and N_2_O, respectively, which are 1, 21, and 310 based on the currently accepted climate change impact factors^34^. Table 2 shows the list of carbon emission coefficients for the input materials for urban green space maintenance.

**Table 2:** Carbon emission coefficients for the maintenance input materials.

Material	Unit	Climate warming factor *α*_*ci*_/kg CO_2_-e	Source
Petrol	kg	0.39	^35^
Diesel	kg	0.37	^35^
Municipal water	m^3^	0	Without
Fertilizer	kg	1.50	^36,37^
Pesticides	kg	0.35	^36^

## Results

### Correlation analysis between the structural characteristics and annual carbon sequestration of the plant community

The LCS, with an average value of 103.96 kg CO_2_-eq/y^−1^, accounted for 89.2% of the carbon storage capacity of the plant community, indicating that leaf photosynthesis contributed the main source of carbon storage in woody plants. SCS contributed the smallest amount of carbon storage in the plant community, and the mean value accounted for 4.75% (an average of 5.54 kg CO_2_-eq/y^−1^) of the total carbon storage value. The average MCE (7.23 kg CO_2_-eq/y^−1^) accounted for approximately 6.20% of the carbon emissions from community carbon sequestration. The standard deviation of LCS was the highest, at 37.76 CO_2_-eq/y^−1^, compared with that of the other carbon storage characteristics, indicating that the leaf carbon storage data were highly discrete and that leaf carbon storage was easily disturbed by other environmental factors. The average carbon sequestration in the 400 m^2^ plant community was 354.99 kg CO_2_-eq/y^−1^; the maximum value reached 116.46 kg CO_2_-eq/y^−1^, and the standard division of 73.53 represented the strong influence of the structural characteristics on the carbon sequestration values (Table 3).

**Table 3:** Carbon storage and emission values for the plant community in the urban green space.

Sequestration type	Min	Max	Average	Sd
LCS kg CO_2_-eq/y^−1^	10.39	300.09	103.96	37.76
BCS kg CO_2_-eq/y^−1^	0.53	55.66	14.18	10.68
SCS kg CO_2_-eq/y^−1^	1.08	10.43	5.54	2.46
MCE kg CO_2_-eq/y^−1^	0.22	27.65	7.23	4.62
CT kg CO_2_-eq/y^−1^	30.39	354.99	116.46	73.53

Carbon storage and emission values in the 400 m^2^ plant community samples. *LCS* leaf photosynthetic carbon storage, *BCS* cumulative plant biomass carbon storage, *SCS* soil carbon storage, *MCE* maintenance carbon emissions, *CT* total carbon sequestration by the plant community. CT was calculated as the sum of LCS, BCS, and SCS minus MCE.

Pearson's linear and nonlinear regression analyses were conducted using MATLAB_R2021a for the correlation analysis between the horizontal (density, coverage, angular scale) and vertical (delta height, height and CCH) structural characteristics of the sample plant communities and their carbon sequestration indicators. The analysis showed that the horizontal structural characteristics exhibited significant correlations with annual carbon sequestration, whereas the vertical structural characteristics did not show any significant correlation (Table 4). Density was significantly related to all carbon sequestration indicators (LCS, BCS, SCS, and MCE; p < 0.05), and coverage was significantly related to LCS, BCS, and SCS. The mean height of the plant community was significantly related only to the MCE, while the remaining vertical structural characteristics showed no significant correlation with the carbon sequestration indicators. Thus, density and coverage were identified as the major structural characteristics that regulated the annual carbon sequestration capacity in the green space plant communities.

**Table 4:** Linear and nonlinear regression analyses of structural characteristics and carbon sequestration indicators of the plant community.

	N	Biomass		Leaves		Soil		Maintenance	
		Linear	Nonlinear	Linear	Nonlinear	Linear	Nonlinear	Linear	Nonlinear
Density	106	0.00091**	0.0071**	0.0004**	0.0028**	0.0015**	0.0016**	0.0001**	0.0018**
Coverage	106	0.0079**	0.56	0.012*	0.16	0.001**	0.012**	0.39	0.07
Angle	106	0.512	0.733	0.89	0.37	0.22	0.22	0.069	0.13
Delta height	106	0.649	0.915	0.79	0.66	0.97	0.87	0.23	0.29
Height	106	0.861	0.789	0.94	0.99	0.93	0.63	0.0003**	0.0006**
CCH	106	0.431	0.749	0.44	0.64	0.22	0.83	0.029	0.03

**Correlation is significant at the 0.01 level, *Correlation is significant at the 0.05 level. Significant values are in bold.

### Regression models of structural characteristics and annual carbon sequestration of the plant community

To verify the accuracy of the model, we fit the model using six models (Weibull, Logistic, Modified logistic, Lundqvist, Gompertz, Chapman–Richards) from the multiple linear regression analysis. MATLAB_R2021a was used to fit six nonlinear regression models to the relationships of the 2 independent variables (density and coverage) of the plant community characteristics and the annual carbon sequestration values of the plant communities^38^. The variables that had a significant effect in the nonlinear model were progressively obtained by comparing the p values of the *t*-tests for each assessed coefficient and further confirming the specific assessed coefficients, as shown in Appendix B (Table A4). In the univariate view, the six nonlinear regression models did not have any weighting functions fitted to the six independent variables, which is consistent with the results of the correlation analysis described above. Under the same weighting function, the different regression equations were compared for AIC, AICC, BIC, and CAIC by choosing the minimum value, and the ordinary and adjusted regressions were based on the maximum value, resulting in the following nonlinear fitted equations: Logistic, Weibull, and Chapman–Richards functions (Table 5).

**Table 5:** The nonlinear regression models of the structural characteristics and carbon sequestration of the plant community.

Regression model	*Y*	*X*	equation	Ordinary	Adjusted
Logistic^38^	Biomass carbon storage	Planting density	${\text{y}} = \frac{22.8}{{1 + 7.94{\text{e}}^{{ - 41.59x_{1} }} }}$	0.27	0.26
Chapman–Richards^39^	Biomass carbon storage	Coverage degree	${\text{y}} = 270\left[ {1 - e^{{ - \left( {0.006x_{2} } \right)}} } \right]^{0.55}$	0.11	0.10
Logistic^38^	Leaf carbon storage	Planting density	${\text{y}} = \frac{207.7}{{1 + 7.16{\text{e}}^{{ - 29.49x_{1} }} }}$	0.46	0.45
Chapman–Richards^39^	Leaf carbon storage	Coverage degree	${\text{y}} = 1647.8\left[ {1 - e^{{ - \left( {0.004x_{2} } \right)}} } \right]^{0.47}$	0.10	0.09
Logistic^38^	Maintenance carbon emissions	Planting density	${\text{y}} = \frac{11.15}{{1 + 1.55{\text{e}}^{{ - 33.04x_{1} }} }}$	0.29	0.27
Weibull^40^	Maintenance carbon emissions	Coverage degree	${\text{y}} = 7.39\left[ {1 - e^{{ - 1.64(x_{2}^{ - 2.95} )}} } \right]$	0.08	0.06
Logistic^38^	Soil carbon storage	Planting density	${\text{y}} = \frac{8.89}{{1 + 3.66{\text{e}}^{{ - 30.24x_{1} }} }}$	0.50	0.49
Chapman–Richards^39^	Soil carbon storage	Coverage degree	${\text{y}} = 127.72\left[ {1 - e^{{ - \left( {0.0058x_{2} } \right)}} } \right]^{0.56}$	0.35	0.34

**Figure 4:**
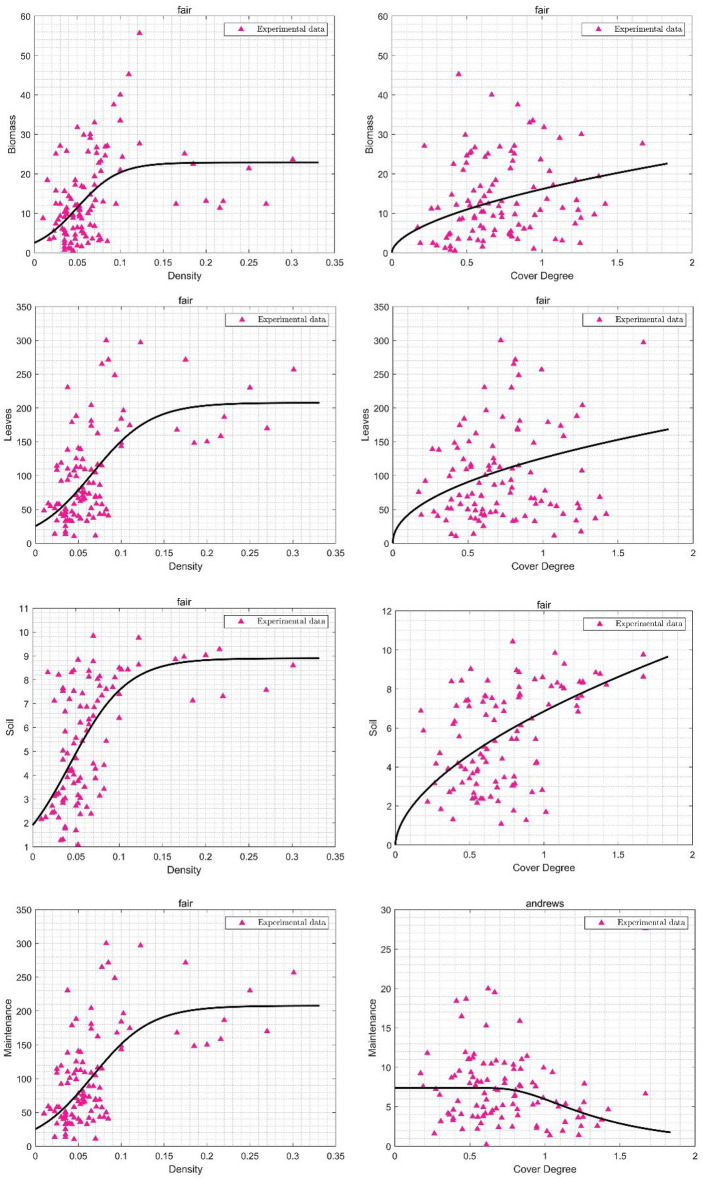
Nonlinear regression analysis of key structural characteristics and carbon sequestration indicators in the plant community. (They-axis in Biomass, Leaves and Soil represents carbon storage value, in kg/CO_2_-eq/y^−1^; and they-axis in Maintenance represents carbon emission value, in kg/CO_2_-eq/y^−1^).

In the plant community, structural characteristics in some specific intervals led to dramatic changes in the carbon sequestration values. For example, an increase in plant density from 0 to 0.15 and the coverage from 0.5 to 1.5 generally indicated a considerable increase in the annual carbon storage capacity of the plant community: the LCS, BCS and SCS reached 167.00 kg/CO_2_-eq/y^−1^, 16,57 kg/CO_2_-eq/y^−1^ and 8.86 kg/CO_2_-eq/y^−1^, which increased by 153.33% (kg/CO_2_-eq/y^−1^), 33.84% (12.38 kg/CO_2_-eq/y^−1^) and 63.16% (5.43 kg/CO_2_-eq/y^−1^), respectively, compared to the values in parentheses. LCS contributed the most to the carbon storage capacity of the plant community, which was approximately 10.12 and 18.9 times higher than the BCS and SCS values, respectively. The increase in plant density and coverage caused an opposite change in the MCE: the increase in density from 0.05 to 0.15 led to a 63.15% increase in the MCE from 70.00 to 190.00 kg/CO_2_-eq/y^−1^; however, the increase in coverage from 0.5 to 1.5 caused a 65.71% decrease in MCE from 11.02 to 6.65 kg/CO_2_-eq/y^−1^. In general, based on the carbon indicator changes according to the structural characteristics, the density in a specific interval (0–0.15) had a greater influence on the change rate of the carbon storage and emission values than the plant community coverage (Fig. 4).

### Nonlinear regression model between structural characteristics and carbon sequestration in the plant community

**Figure 5:**
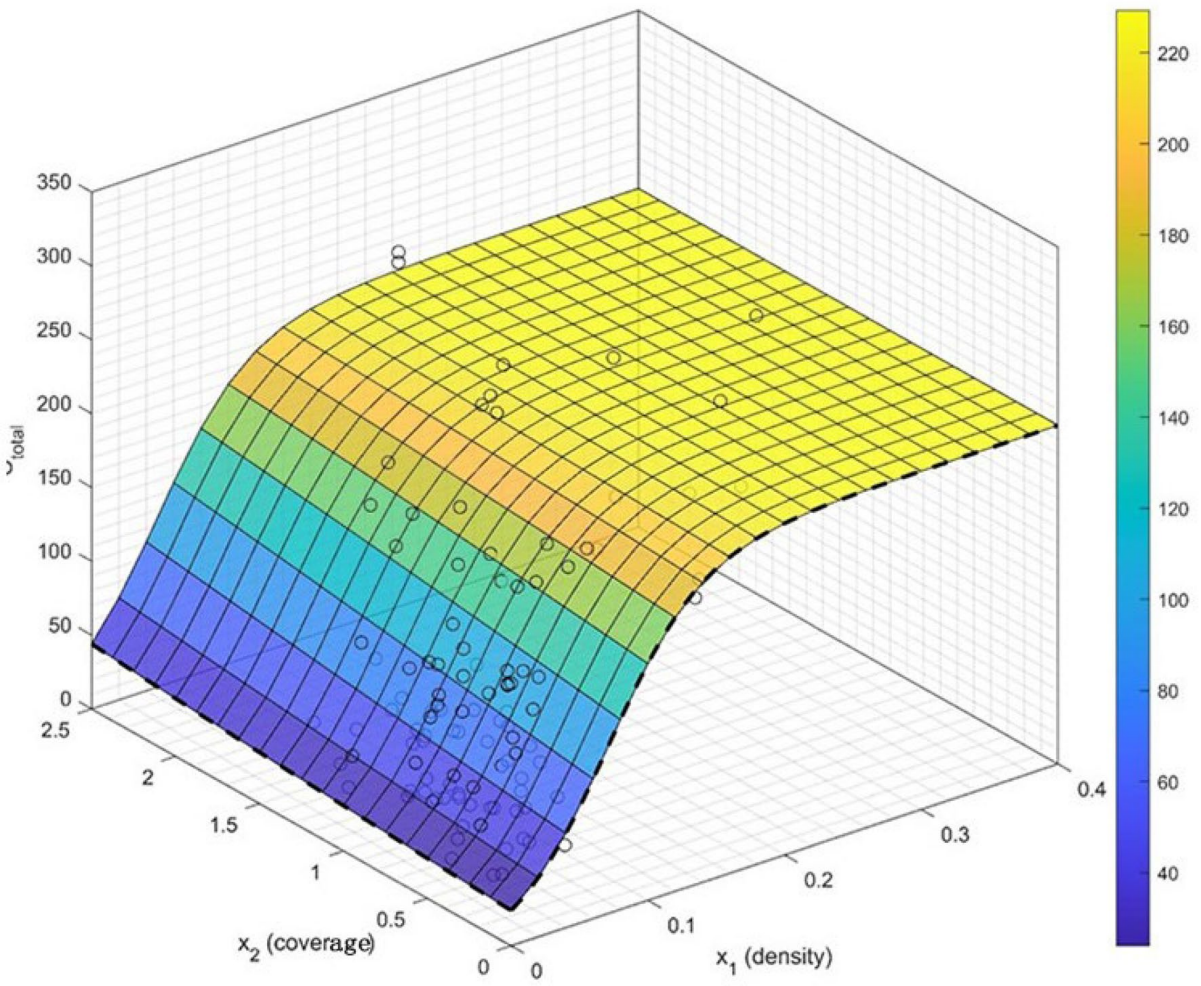
Three-dimensional plot of the nonlinear regression model of the structural characteristics and carbon sequestration of the plant community. (They-axis represents carbon sequestration value, in kg/CO_2_-eq/y^−1^).

Based on a comparison of the magnitude of the information criterion AIC (AICc for multivariate nonlinear regression models, as shown in Appendix C in bold (Table A5), taking into account the significant characteristics of each estimated coefficient, the following statistical model was determined to predict the integrated carbon sequestration with changes in the density (X_1_) and coverage (X_2_) of the plant communities.

13 $$\begin{array}{*{20}c} {C = \frac{229.31}{{1 + 8.57e^{{ - \left( {29.89x_{1} + 0.2835x_{2} } \right)}} }}} \\ \end{array}$$

where C is the total carbon sequestration of the plant communities, in kg/CO_2_-eq/y^−1^. The three-dimensional plot for the regression model is shown in Fig. 5.

The fitted equations showed that the degree of influence of the plant density and coverage on the integrated carbon sequestration of the community was as high as 43% (the fair weight function was 0.4348). Density and coverage can be adjusted to increase the carbon sequestration capacity of the plant community in urban greenspaces. The carbon sequestration result of the three-dimensional plot representing the visualization of the nonlinear regression model shows that the density has a significant effect on the carbon sequestration capacity, and the improvement in the carbon sequestration capacity will reach a stable status when the density exceeds 0.15 (Fig. 5). To understand the strength of the influence of each independent structural characteristic variable (density and coverage) on the carbon sequestration model, the same weight function was fitted to the nonlinear equation by normalizing the z score:

14 $$\begin{array}{*{20}c} {\tilde{C} = \frac{1}{{2e^{{ - 12.43\widetilde{{x_{1} }} + 0.4563\widetilde{{x_{2} }}}} }}} \\ \end{array}$$

The z score equation shows that density has an effect that is 27.24 times stronger than that of coverage in the regulating effect on carbon sequestration in the plant communities.

## Discussion

### Influence of structural characteristics on carbon sequestration indicators in the plant community

There was a significant positive correlation between density and coverage and the aboveground carbon storage capacity (LCS and BCS) in the plant community. The density increases with species diversity, which has a direct effect on biomass changes^41,42^. The carbon sequestration capacity of bamboo forests is independent of environmental factors but increases with density, implying that density is the crucial factor that affects carbon sequestration in plant communities^43^. Higher densities increases plant photosynthesis, above-and belowground biomasses, which in turn increase community carbon sequestration. Simultaneously, plant communities with higher coverage can promote the efficiency of light absorption to enhance the photosynthetic capacity and improve the carbon sequestration capacity of the plant community^44^. However, although density is positively correlated with the carbon sequestration capacity, as a growth process, a higher plant density can compress the growth space and increase the mortality risk of individual plants in the community, impacting plant biomass accumulation^5^. On the other hand, a continual increase in density will cause the overlapping of plant crowns and will influence the community photosynthetic capacity, leading to an increase in plant disease risk^45^. The changes in LCS and BCS at a density higher than 0.15 in this study support the above views, i.e., a density that is too high will influence the carbon sequestration of the plant community (Fig. 4).

The significant positive correlation between density and coverage with SCS in the plant community is consistent with the findings of previous research. Lange noted that increasing plant density and biodiversity increased carbon input from roots to microbial communities, resulting in the improvement of soil microbial activity and the carbon storage capacity^46^. An increase in plant density implies an increase in the amount of leaf apoptosis and subsurface roots, which are the main source of soil organic carbon, and decomposition not only increases the carbon content of the soil but also improves the physicochemical properties of the soil. Moreover, improving belowground roots by increase the density can positively affect the rate of soil carbon accumulation and thus alter soil activity^47^. Livesley measured higher soil carbon storage values in areas where trees had been planted than in a nearby grassland in golf courses^48^. In addition, an increase in plant coverage usually implies a decrease in the bare surface, which can reduce the erosion due to rainfall and the loss of organic carbon from the soil surface. In conclusion, an increase in the plant density and coverage improved soil vigour, resulting in an improvement in the soil carbon capacity.

The density and average height were significantly and positively correlated with the MCE. Strohbach showed that 54% of MCE was attributed to pruning and the removal of woody plant litter, 22% to transportation and 24% to grassland maintenance, suggesting that pruning has the greatest impact on MCE^49^, and that higher density plant communities require high frequency of pruning efforts to maintain the ecological function of plant communities, which further supports the view of this paper that density is the main factor influencing MCE. Plant communities with higher average heights require special equipment and techniques for maintenance, such as high branch pruning, in addition to taller trees requiring more water, fertilizer, and other inputs to support their growth^50^. Although plant communities with higher densities and average heights produce more MCE, these plant communities also provide more ecological functions (e.g., carbon sequestration), which requires us to find the best management strategies to minimize MCE while maximizing the ecological functions of plant communities during planning and management.

### Influence of plant community structural characteristics on total carbon sequestration

Density played a key structural role in regulating the total carbon sequestration of the plant communities, being 27.24 times more effective than coverage. The reason for the high variability between the two is that density is a reflection of the number of individual plants per unit area, and all other things being equal, higher densities will directly increase biomass and carbon sequestration. Although coverage is an important ecological parameter, if coverage is increased, biomass and carbon sequestration will still be lower if individual plants are smaller. On the other hand, high-density plant communities contribute more to soil carbon sequestration through their root systems and leaf litter, as more plant residues are incorporated into the soil, thus contributing to soil carbon sequestration capacity. At this point, density plays a greater role than coverage. It suggests that increasing the density of plant communities is an effective strategy to increase carbon storage capacity. In addition, in urban green space design, it is recommended that the woody planting area does not exceed 70% of the plant community; otherwise, it will affect the living space of plants, which will also increase the daily workload of maintenance tasks and produce a higher MCE^50^. Thus, the improvement of plant density and coverage has a positive effect on increasing carbon sequestration, but there are also certain limitations. The regulation of density on carbon sequestration has been the focus of ecologists' attention, and it is important in the choice of a suitable density as well as the restoration and improvement of the ecological function of urban environments with high carbon emissions^51^.

### Regulation of the structural characteristics to improve carbon sequestration in the plant community

**Figure 6:**
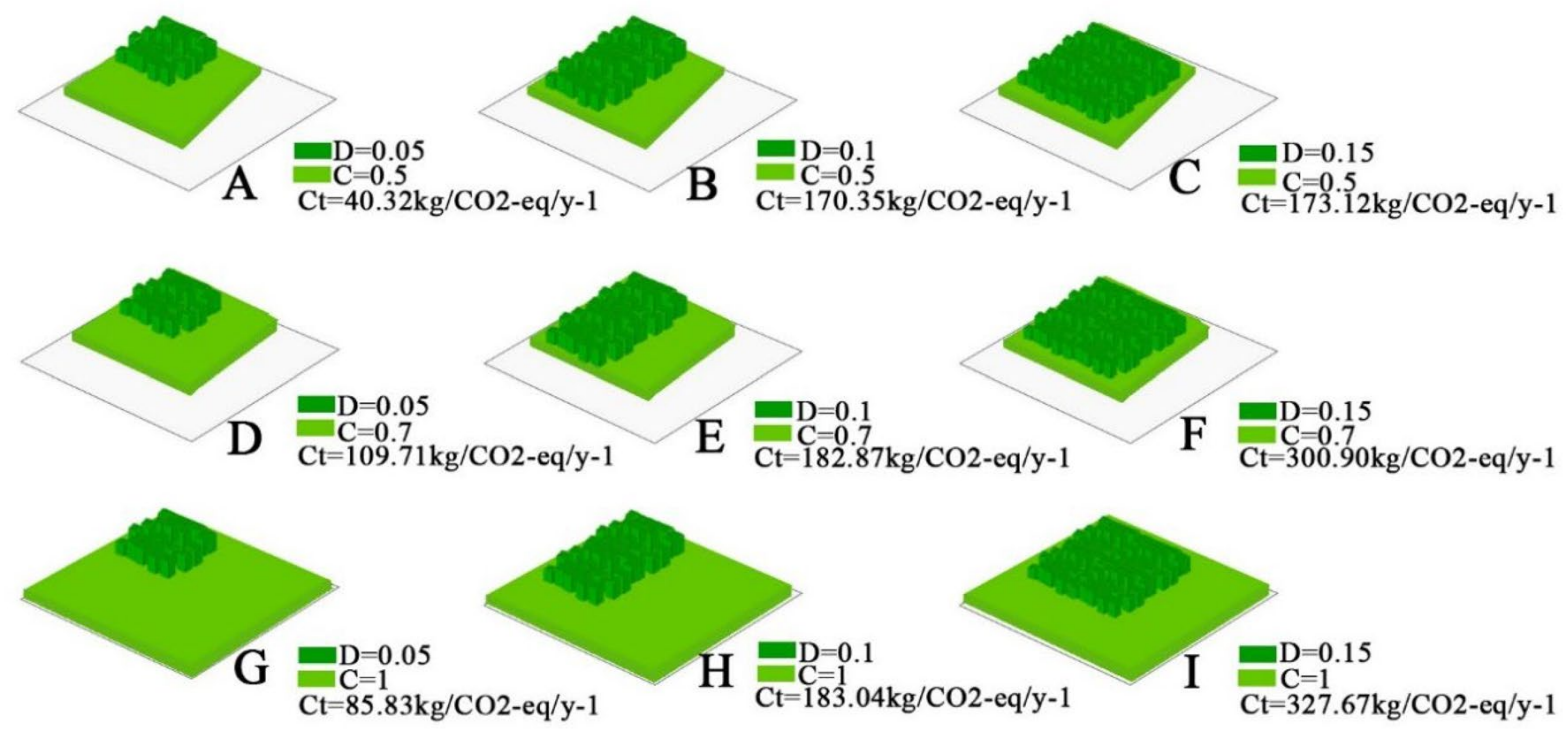
Strategy for regulating plant community characteristics. (where D is density, C is coverage, and Ct is the carbon sequestration value, in kg/CO_2_-eq/y^−1^. The sample plot area was 400 m^2^).

The purpose of regulating the structural characteristics of plant communities is to increase the carbon sequestration capacity and to ensure the maximization of multiple ecological benefits. When the density and coverage were 0.15 and 1, respectively, the carbon sequestration reached the optimal value of 327.67 kg CO_2_-eq/y^−1^ in the 400 m^2^ plant community (Fig. 6). Although the carbon sequestration continued to increase thereafter with increasing density and coverage, up to 333.54 kg CO_2_-eq/y^−1^ within the community sample, it was only 1.7% higher than the optimal value, which may also have negative effects on the development of other ecological benefits of the community. Li's study suggested that too high a community density (D ≥ 0.15) may lead to the problem of declining biodiversity^52^. The reasons are as follows: at high density, light-loving species need sufficient sunlight to grow well, and shorter plants are limited by light and growing space, and their survival rate is lower (D ≥ 0.15). He showed that a community with a high density (D ≥ 0.15) is prone to pests and diseases, and when the community density is too high, the stressed wood in the community is often a settlement site for certain pathogens, which can easily form infestation centres and jeopardize the health of the community^53^.

In addition, when the density and cover are 0.15 and 1, the community structural ecological functions of cooling and humidification, wind and sand control, and dust retention can be brought into play. At these values, the community canopy is closed, which can result in effective blockage of the solar radiation intensity and wind and sand invasion, and the uniformity, coherence, and sparseness of the plant leaf arrangement can effectively strengthen the dust retention function of the plant community^54,55^. Therefore, we believe that when the density and cover are 0.15 and 1, respectively, the plant community sequesters carbon to reach the optimal value, and at the same time meets a variety of ecological functions such as low pest and disease rates, cooling and humidification, and wind and sand control. The plant community is a significant place to ensure that the composite ecological functions of the urban area are performed together; its carbon sequestration function should be enhanced, and other ecological functions should be performed simultaneously. When constructing plant communities with high carbon sequestration capacity, it is necessary to ensure that the density and coverage characteristics of the community are within a reasonable range to promote the full play of the multiple ecological functions of green spaces. In addition, density and cover are key factors affecting forestry management and forest ecosystems. Proper control of density and cover is essential for forest restoration and regeneration, and scientific management can help restore damaged forests and promote ecological balance. Overall, optimizing density and cover through scientific forestry management is an important strategy for maintaining forest health and productivity. We investigated the carbon sequestration capacity of different tree species in our article, but the focus of this study is on the relationship between plant community structure special diagnosis and carbon storage, so we did not focus and discuss the carbon sequestration capacity of different tree species in our study.

## Conclusions

The structural characteristics had a strong influence on the carbon sequestration capacity of the plant community in the urban green space. Density and coverage were significantly and positively correlated with aboveground and soil carbon storage (LCS, BCS and SCS). Density and mean height were significantly and positively correlated with MCE. Density played a key structural role in regulating the total carbon sequestration of the plant communities, being 27.24 times more effective than coverage. The community carbon sequestration was optimal at 327.67 kg CO_2_-eq/y^−1^ for density and coverage values of 0.15 and 1, respectively. We consider the density, cover in this range to be optimal for annual carbon sequestration by the plant community.

There are still many uncertainties about the effects of plant community structural characteristics on aboveground and soil carbon storage and maintenance carbon emissions, such as how the soil conditions and aboveground vegetation interact with each other and thus act on the formation and transformation of soil carbon pools. These areas remain the focus of research in the field of urban ecology. In this paper, we summarize the key structural characteristics that regulate the carbon sequestration capacity of plant communities, analyse the statistical relationships between structural characteristics and carbon indicators, and propose a recommended range of structural characteristics with the aim of improving the total carbon sequestration and respecting the multiple eco-functions in the plant community of urban green spaces. This paper provides a quantitative reference for the planting and maintenance of urban green spaces and promotes the enhancement of carbon sequestration and the development of sustainable green spaces in urban ecosystems (all data generated or analyzed during the course of this study are included in this article or supplemental data).

### Supplementary Information

Supplementary Information.

Supplementary Tables.

## Data Availability

The datasets used and/or analyzed during the current study available from the corresponding author on reasonable request.
