# Supplementary material for: Effects of plant community structural characteristics on carbon sequestration in urban green spaces
Source: Sci Rep. 2024 Mar 28;14:7382. doi: 10.1038/s41598-024-57789-2 (PMC10978906; doi:10.1038/s41598-024-57789-2)
Supplement: Supplementary file 1 — Supplementary Information. [file 41598_2024_57789_MOESM1_ESM.pdf]

	leaves(kg/year)	biomass(kg/year)	soil（Kg）
yangfang1	265.0539672	23.26183158	7.14
yangfang2	71.04161434	3.50770496	4.32
yangfang3	88.91400672	11.8284262	6.38
yangfang4	51.22066118	4.861858176	3.31
yangfang5	73.23193786	25.66377619	5.66
yangfang6	88.85869978	10.05427434	5.86
yangfang7	114.7650578	3.413148005	7.34
yangfang8	230.591592	10.40007578	3.67
yangfang9	57.79810714	15.73139515	3.12
yangfang10	57.88663373	8.420265339	3.19
yangfang11	13.82380877	5.524374664	2.43
yangfang12	36.06522797	9.185277322	4.76
yangfang13	143.4376944	40.03949013	8.40
yangfang14	104.3884195	32.98370136	6.48
yangfang15	114.4619562	25.07422726	2.46
yangfang16	180.8362322	7.095239095	6.13
yangfang17	125.2797638	17.18095074	4.32
yangfang18	124.163424	8.633369966	4.88
yangfang19	184.1863605	20.95179986	8.40
yangfang20	112.6569629	11.98816213	4.36
yangfang21	99.19471162	4.551133551	4.39
yangfang22	174.5920915	45.24527289	8.43
yangfang23	108.3334856	12.43288821	2.46
yangfang24	48.45722112	8.762804885	2.16
yangfang25	10.3938912	0.533923027	4.19
yangfang26	13.52647296	0.716196267	3.64
yangfang27	271.5174996	27.11406749	7.43
yangfang28	58.05792115	9.563995449	4.25
yangfang2	107.411851	8.820518652	4.31
yangfang3	40.81235558	2.899492832	7.61
yangfang5	162.4509976	26.68308339	6.87
yangfang6	69.60860122	6.779400679	6.48
yangfang7	139.2871899	11.20404361	4.76
yangfang8	62.11745971	10.84039195	4.82
yangfang9	36.83761805	9.711360963	4.84
yangfang10	296.8945165	55.66925966	8.63
yangfang11	173.6188312	29.12626423	6.13
yangfang12	167.7680484	12.38081951	8.09
yangfang13	57.3893591	20.70031928	7.14
yangfang14	37.39255027	3.571510078	6.38
yangfang15	82.03598726	5.452531529	5.05
yangfang16	196.3952686	24.27398107	8.43
yangfang17	40.03358515	6.118151058	3.27
yangfang18	46.59088896	2.492447398	4.17
yangfang19	140.6744064	12.14529991	4.56
yangfang20	91.97895168	27.07635836	3.22
yangfang21	118.8174113	9.304189462	3.24
yangfang22	58.90351104	18.4243666	2.24
	75.72445056	6.403451054	4.88
	42.08888218	6.155290522	4.25
	33.5869079	1.20725568	3.91
	51.56262144	3.765389004	2.71
	41.90427648	2.439186573	5.86
	109.2357446	29.86384307	5.43
	100.8286583	14.33644438	3.91
	54.89571226	3.388518552	2.31
	138.3245614	11.37869943	3.83

43.33238554	3.070852365	7.03
64.59640704	1.638685185	5.21
300.0976957	26.87006167	7.42
66.62037888	6.420720137	4.71
116.3543547	25.25030211	6.87
248.6319391	37.54306739	7.69
68.40994406	19.38551784	6.77
50.05863245	24.61858481	7.42
77.56632576	31.80419024	4.69
178.9300973	13.6268035	4.19
68.82378624	22.7488458	7.11
188.226644	18.41506069	4.53
70.4419753	16.86813306	5.36
49.79533363	10.32281473	3.64
112.5361797	11.76369059	4.86
33.00115738	3.17624524	3.65
11.17948677	17.17228146	6.84
204.1365681	30.02503944	6.37
74.43198259	4.948436812	5.43
108.7131387	14.70400468	6.34
40.25742336	1.855180235	4.71
65.92537037	16.57582904	5.43
25.3984896	1.535744605	3.65
110.0342707	25.75595209	3.76
51.90461338	10.84900368	6.37
34.06294426	9.744454787	3.83
33.3592681	7.378261778	3.12
46.65528806	5.730219313	3.65
17.40737434	2.433247059	3.64
32.58168883	1.029649071	4.19
41.25906893	8.996915864	3.46
86.28050534	4.409880784	7.11
93.46879872	4.601591792	3.83
32.9370624	4.114168813	5.51
271.5174996	25.11406749	8.96
148.2494872	22.52066157	9.13
296.8945165	27.66925966	8.76
167.7680484	12.38081951	8.86
148.4319391	33.54306739	8.19
58.32544064	13.35178386	6.84
43.05585245	12.92858481	7.34
43.3592681	12.37826178	3.21
45.65528806	15.62302193	3.53
230.1359958	21.4090414	9.43
150.2456872	13.1573284	9.23
256.7645165	23.66925966	9.6
169.9804845	12.38081951	9.57
186.6193907	13.06736611	9.31
158.4254406	11.37830086	9.28

maintenance	total
7.132749	288.3230498
4.07068725	74.79863205
8.15068725	98.97174567
3.289658	56.10286136
11.66068725	92.8950268
10.5713745	94.20345289
9.802749	115.7154568
0.225	244.4366678
5.14068725	71.50881504
2.149658	67.34724107
7.702749	14.07543443
8.25206175	41.75844354
19.522749	172.3524145
8.062749	135.7893719
4.23	137.7661835
11.0213745	183.0400968
7.264658	139.5160566
11.902749	125.774045
18.682749	194.8533904
8.759658	120.245467
8.689658	99.44618717
16.47843625	211.7889282
10.45343625	112.7729375
2.20068725	57.17933875
5.719658	9.394984343
4.639658	13.24301123
4.684658	301.3769091
5.5	66.3719166
5.5713745	114.9709952
15.3	36.02184842
3.7099245	192.2941565
10.4813745	72.3866274
1.62	153.6312335
6.1313745	71.64647716
2.589316	48.79966301
27.6563745	333.5374016
1.95	206.9250955
15.888247	172.3552246
9.3713745	75.85830388
3.77068725	43.5733731
9.76568725	82.77219697
19.979658	209.1182281
11.419658	38.00207821
7.249658	46.00367836
9.5413745	147.8383318
11.812749	110.462561
10.8413745	120.5202263
2.4202749	77.14760274
9.2513745	77.75652711
5.899658	46.5945147
4.174658	34.52950558
4.0013745	54.03663594
7.564658	42.64065833
11.032749	133.4968387
8.407749	110.6673537
5.00068725	55.59354356
3.17068725	150.3625736

3.075	50.3582379
3.53068725	67.91044435
5.19568725	329.1920702
5.2613745	72.48972452
11.2313745	137.2432823
7.6013745	286.263632
3.349658	91.2158039
7.969658	74.12755926
1.89	112.170516
1.38	195.3637289
11.06068725	87.62194479
1.425	209.7467047
3.83068725	88.83942111
6.71068725	57.04746112
3.604658	125.5552123
2.91	36.91740262
5.57068725	29.62108099
7.94068725	232.5909203
2.46	82.3504194
8.94	120.8171434
6.499658	40.3229456
2.299658	85.63154141
4.159658	26.42457621
6.35068725	133.1995356
2.539658	66.58395906
3.919658	43.71774104
3.66	40.19752987
7.1213745	48.91413288
3.72	19.7606214
2.54068725	35.25747877
8.5313745	45.18461029
4.789658	93.01072813
5.3213745	96.57901601
6.48	36.08130915
4.684658	300.9069091
18.4213745	161.4787743
6.6563745	326.6702934
15.888247	173.120621
7.59013745	182.5794726
4.649658	73.8675665
4.959658	58.36477926
4.666528	54.28100187
4.5216745	60.2866355
10.333486	250.6415512
9.374565	163.2584506
9.9395	280.0942761
7.73598	184.195324
8.013988	200.9827688
5.3658	173.7179415
