# Supplementary material for: Effects of plant community structural characteristics on carbon sequestration in urban green spaces
Source: Sci Rep. 2024 Mar 28;14:7382. doi: 10.1038/s41598-024-57789-2 (PMC10978906; doi:10.1038/s41598-024-57789-2)
Supplement: Supplementary file 2 — Supplementary Tables. [file 41598_2024_57789_MOESM2_ESM.docx]

# Appendix:

# Appendix A

**Table A1** Photosynthetic data of individual tree species

| Tree species | P/mmol/m² | WCO_2_/g/m²·d | QCO_2_/g/m²·d |
| --- | --- | --- | --- |
| Prunus salicina Lindl. | 351.36 | 15.45 | 41.27 |
| Koelreuteria paniculate Laxm. | 477.72 | 21.01 | 71.88 |
| Sabina chinensis（L.）Ant.(Juniperus chinensis L.) | 136.44 | 6.00 | 8.16 |
| Acer palmatum Thunb. | 148.32 | 6.52 | 11.61 |
| Cedrus deodara (Roxb.) Loud. (C.libani Rich. Var. deodara Hook. f.) | 251.28 | 11.05 | 23.21 |
| Catalpa bungee C. A. Mey. | 350.28 | 15.41 | 39.30 |
| Magnolia grandiflora L. | 409.68 | 18.02 | 45.42 |
| Buxus sinica (Rehd. et Wils.) Cheng (B.microphylla Sied .et Zucc.var sinica Rehd .et Wils) | 243.72 | 10.72 | 14.26 |
| Platanus orientalis L. | 387.36 | 17.04 | 40.90 |
| Nerium indicum Mill. | 96.12 | 4.22 | 4.22 |
| F．nubro-plena Schneid. | 249.84 | 10.99 | 21.32 |
| Cinnamomum camphora (L.) Presl | 388.8 | 17.10 | 53.88 |
| Lagerstroemia indica L. | 195.12 | 8.58 | 9.44 |
| Ilex cornuta Lindl. | 45.72 | 2.01 | 1.52 |
| Prunus mume Sieb. et Zucc. | 69.12 | 3.04 | 3.40 |
| Photinia serrulate Lindl. | 146.16 | 6.43 | 3.98 |
| Eriobotrya japonica (Thunb.) Lindl. | 76.32 | 3.35 | 7.11 |
| Hibiscus syriacus L. | 216 | 9.50 | 14.73 |
| Punica granatum L. | 159.84 | 7.03 | 10.05 |
| Malus micromalus Mak | 176.76 | 7.77 | 17.88 |
| Nandina domestica Thunb. | 129.96 | 5.71 | 2.63 |
| Pittosporum tobira（Thunb.）Ait. | 266.4 | 11.72 | 13.01 |
| Rosa chinensis Jacq. | 129.96 | 5.71 | 2.40 |
| Cycas revoluta Thunb. | 147.6 | 6.49 | 6.94 |
| Gleditsia sinensis Lam. | 151.2 | 6.65 | 11.24 |
| Osmanthus fragrans （Thunb.）Lour. | 293.04 | 12.89 | 24.75 |
| Ginkgo biloba L. | 204.12 | 8.98 | 15.44 |
| Ulmus pumila L. | 308.88 | 13.59 | 33.02 |
| Ligustrum lucidum Ait. | 48.6 | 2.13 | 3.20 |
| Pinus bungeana Zucc. | 276.48 | 12.16 | 10.34 |
| Sophora japonica L. | 330.78 | 11.64 | 25.38 |
| Salix babylonica L. | 400.45 | 14.09 | 28.05 |
| Syringa oblata Lindl. | 50.6 | 1.78 | 2.58 |
| Jasminum nudiflorum Lindl. | 100.75 | 3.54 | 6.59 |

**Table A2** Biomass models for different tree species

| Tree species | Biomass model | DBH/cm | R² |
| --- | --- | --- | --- |
| Elaeocarpus sylvestris （Lour.）Poir. | 0.18448D^1.93050^+  0.11932D^1.94825^+  0.02792D^2.12843^+  0.07138D^1.10470^ | 2.10-16.62 | 0.98  0.99  0.93  0.56 |
| Ligustrum lucidum Ait | 0.47118D^1.64813^+  0.18954D^1.86853^+  0.13282D^1.47593^+  0.24793D^0.89346^ | 2.89-16.80 | 0.98  0.99  0.81  0.62 |
| Koelreuteria paniculate Laxm. | 0.12238D^2.13793^+  0.11478D^2.06573^+  0.00111D^3.20022^+  0.02068D^1.68502^ | 3.00-17.9 | 0.95  0.97  0.88  0.73 |
| Sapindus mukurossi Gaertn | 0.14119D^2.35753^+  0.12488D^2.09316^+  0.01439D^2.92804^+  0.03200D^1.98218^ | 2.19-18.00 | 0.97  0.98  0.95  0.87 |
| Cinnamomum camphora (L.) Presl | 0.10387D^2.53500^+  0.03345D^2.43692^+  0.07086D^2.27885^+  0.02302D^1.93423^+  0.01141D^2.85885^+  0.00139D^3.23231^ | 7-17 | 0.99  0.96  0.97  0.81  0.94  0.79 |
| Liriodendron chinense (Hemsl.) Sarg. （L.tulipifera var. chinense Hemsl. | 0.06393D^2.61147^+  0.04772D^2.10647^+  0.01959D^2.80941^+  0.00321D^2.83529^+  0.00715D^2.85853^ | 4-14 | 0.97  0.93  0.98  0.96  0.89 |
| Bischofia polycarpa (Levl.) Airy Shaw（B.racemosa Cheng et C.D.Chu） | 0.03098D^2.74623^+  0.01001D^2.81178^+  0.02262D^2.85432^+  0.00475D^3.10020^+  0.00005D^4.03846^+  0.00003D^4.17661^ | 4-15 | 0.97  0.99  0.99  0.97  0.97  0.97 |
| Populus L. | 0.019011D^3.1051^+  0.013449D^2.4535^+  0.006725D^3.1964^+  0.001848D^3.0384^+  0.003399D^2.6815^+  0.001885D^3.0213^ | 5-25 | 0.995  0.984  0.990  0.982  0.961  0.987 |
| Metasequoia glyptostroboides Hu et Cheng | 0.06291D^2.4841^+  0.02163D^2.7593^+  0.02998D^2.0946^+  0.10842D^1.3673^ | 2.51-3.04 | 0.972  0.950  0.956  0.917 |
| Bischofia javanica Bl. | 0.1815D^2.3222^ | 5-20 |  |
| Cycas revoluta Thunb. | 1590.5e-0.112D |  |  |
| Pittosporum tobira（Thunb.）Ait. | 0.0043(D^2^H)^0.9204+0.0244^（D2H）^0.8332^ |  |  |
| Ficus lacor Buch.-Ham | 0.1815D^2.3222^ | 5-20 |  |

**Table A3** Biomass models for different diameter classes

| DBH | Regression equation | Adj·R² | SEM |
| --- | --- | --- | --- |
| D≤5 cm | WT=0.05549×D^2.87776^+  WB=0.01124×D^3.16237^+  WL=0.01551×D^2.32693^+  WR=0.02838×D^2.65348^+ | 0.91164  0.81933  0.86555  0.90495 | 0.60826  0.30284  0.08602  0.22077 |
| 5＜D≤10 cm | WT=0.11701×D^2.36933^+  WB=0.01621×D^2.93859^+  WL=0.04169×D^1.90082^+  WR=0.04977×D^2.19517^+ | 0.88428  0.76490  0.68922  0.95730 | 2.05700  1.79321  0.44047  0.32819 |
| 10≤D≤20 cm | WT=0.10769×D^2.34891^+  WB=0.00385×D^3.15093^+  WL=0.00372×D^2.65113^+  WR=0.03538×D^2.29567^+ | 0.77761  0.88184  0.82848  0.81687 | 4.15734  3.81171  0.96151  3.46518 |
| D＞20 cm | WT=0.03541×D^2.65146^+  WB=0.00583×D^2.94383^+  WL=0.07709×D^1.55399^+  WR=0.01128×D^2.67850^+ | 0.97844  0.84965  0.71000  0.92962 | 36.71034  52.85291  4.94167  24.5010 |

Note: WT is the trunk biomass; WB is the branch biomass; WL is the leaf biomass; WR is the root biomass. In this paper, only trunk, branch and root biomass models were included, and the leaf component was quantified using the photosynthetic rate

# Appendix B

|  |  | Linear | | Nonlinear | |
| --- | --- | --- | --- | --- | --- |
|  | N | ρ | P | ρ | P |
| Density-Biomass | 106 | 0.3174 | 0.0009 | 0.2992 | 0.0071 |
| Density-Leaves | 106 | 0.5597 | 0.0004 | 0.3700 | 0.0028 |
| Density-Soil | 106 | 0.5450 | 0.0015 | 0.6023 | 0.0016 |
| Density-Maintenance | 106 | 0.3517 | 0.0001 | 0.2855 | 0.0018 |
| Coverage degree- Biomass | 106 | 0.2566 | 0.0079 | 0.1585 | 0.5653 |
| Coverage degree- Leaves | 106 | 0.2243 | 0.0123 | 0.0926 | 0.1605 |
| Coverage degree-Soil | 106 | 0.2243 | 0.0011 | 0.3632 | 0.0128 |
| Coverage degree- Maintenance | 106 | -0.0827 | 0.3988 | -0.1184 | 0.0723 |
| Angle-Biomass | 106 | 0.0642 | 0.5129 | 0.0233 | 0.7336 |
| Angle-Leaves | 106 | 0.0125 | 0.8985 | 0.0611 | 0.3710 |
| Angle-Soil | 106 | -0.1192 | 0.2235 | -0.0823 | 0.2283 |
| Angle-Maintenance | 106 | 0.1767 | 0.0698 | 0.1011 | 0.1384 |
| Delta height-Biomass | 106 | -0.0446 | 0.6496 | -0.0071 | 0.9152 |
| Delta height-Leaves | 106 | -0.0255 | 0.7947 | -0.0284 | 0.6682 |
| Delta height-Soil | 106 | -0.0029 | 0.9762 | -0.0102 | 0.8784 |
| Delta height-Maintenance | 106 | 0.1154 | 0.2384 | 0.06939 | 0.2932 |
| Height-Biomass | 106 | -0.0171 | 0.8615 | 0.0178 | 0.7890 |
| Height-Leaves | 106 | 0.0065 | 0.9472 | 0.0008 | 0.9912 |
| Height-Soil | 106 | 0.0082 | 0.9333 | 0.03169 | 0.6328 |
| Height-Maintenance | 106 | -0.3394 | 0.0003 | -0.2642 | 0.0006 |
| CCH-Biomass | 106 | -0.0771 | 0.4318 | -0.0212 | 0.7494 |
| CCH-Leaves | 106 | -0.0747 | 0.4462 | -0.0305 | 0.6445 |
| CCH-Soil | 106 | -0.1178 | 0.2287 | -0.0143 | 0.8335 |
| CCH-Maintenance | 106 | -0.2113 | 0.0296 | -0.1371 | 0.0375 |

**Table A4**Structural characteristics of plant communities in relation to biomass, leaves, soil and maintenance.

Note: ρ greater than 0.01 indicates significance, p greater than 0.05.

# Appendix C

**Table A5** The estimated coefficients of multivariable nonlinear regressions with different weight functions and the corresponding information criteria.

|  | Weight Function | alpha | gamma | beta1 | AIC | AICc | BIC | CAIC | Ordinary | Adjusted |
| --- | --- | --- | --- | --- | --- | --- | --- | --- | --- | --- |
| Logistic | Andrews | 21.54104202 | 6.298154513 | 40.0407934 | 783.2088 | 783.4441 | 791.1991 | 794.1991 | 0.212471 | 0.19718 |
|  | Bisquare | 21.59773866 | 6.31042777 | 39.95079496 | 783.1692 | 783.4045 | 791.1595 | 794.1595 | 0.212916 | 0.197633 |
|  | Cauchy | 22.09226333 | 7.017225143 | 40.58434149 | 782.7583 | 782.9936 | 790.7486 | 793.7486 | 0.232708 | 0.21781 |
|  | Fair | **22.88393874** | **7.947873768** | **41.5968033** | **782.0453** | **782.2806** | **790.0356** | **793.0356** | **0.277788** | **0.263764** |
|  | Huber | 22.21626339 | 7.308485485 | 41.39399333 | 782.7117 | 782.947 | 790.702 | 793.702 | 0.221379 | 0.20626 |
|  | Logistic | 22.32433843 | 7.117276094 | 40.249679291 | 782.6355 | 782.8708 | 790.6258 | 793.6258 | 0.239715 | 0.224952 |
|  | Talwar | 22.46108046 | 6.372046355 | 40.243710577 | 782.585 | 782.8203 | 790.5754 | 793.5754 | 0.198929 | 0.183374 |
|  | Welsch | 21.78113437 | 6.620690604 | 40.337343113 | 783.0015 | 783.2368 | 790.9918 | 793.9918 | 0.220364 | 0.205226 |
| Chapman-Richards | Andrews | 133.9275 | 0.47090 | 0.010725 | 802.0538 | 802.2891 | 810.0441 | 813.0441 | 0.082028 | 0.064204 |
|  | Bisquare | 131.4814 | 0.470029 | 0.011056 | 802.0815 | 802.3168 | 810.0718 | 813.0718 | 0.081738 | 0.063908 |
|  | Cauchy | 220.938 | 0.501616 | 0.005297 | 801.9101 | 802.1454 | 809.9004 | 812.9004 | 0.091907 | 0.083175 |
|  | Fair | **270.0258** | **0.558035** | **0.006445** | **802.2487** | **802.484** | **810.239** | **813.239** | **0.116524** | **0.108029** |
|  | Huber | 136.1725 | 0.446964 | 0.00818 | 802.5297 | 802.765 | 810.5201 | 813.5201 | 0.072304 | 0.054291 |
|  | Logistic | 202.4181 | 0.514968 | 0.007247 | 801.9325 | 802.1678 | 809.9229 | 812.9229 | 0.096399 | 0.078853 |
|  | Talwar | 16.04781 | 1.438231 | 3.58389 | 805.7367 | 805.972 | 813.727 | 816.727 | 0.054352 | 0.03599 |
|  | Welsch | 148.0508 | 0.480368 | 0.009571 | 802.0407 | 802.276 | 810.031 | 813.031 | 0.084841 | 0.067071 |
| Logistic | Andrews | 208.3098 | 6.563601 | 27.86155 | 1163.604 | 1163.839 | 1171.594 | 1174.594 | 0.433695 | 0.422699 |
|  | Bisquare | 208.314 | 6.557271 | 27.84151 | 1163.621 | 1163.856 | 1171.611 | 1174.611 | 0.433518 | 0.422518 |
|  | Cauchy | 207.546 | 6.715716 | 28.39226 | 1163.602 | 1163.837 | 1171.592 | 1174.592 | 0.440385 | 0.429518 |
|  | Fair | **207.7892** | **7.16232** | **29.49718** | **1163.009** | **1163.244** | **1170.999** | **1173.999** | **0.461368** | **0.450909** |
|  | Huber | 208.5374 | 6.316344 | 27.32314 | 1164.037 | 1164.272 | 1172.027 | 1175.027 | 0.417431 | 0.406119 |
|  | Logistic | 207.3959 | 6.818346 | 28.70516 | 1163.464 | 1163.699 | 1171.454 | 1174.454 | 0.44404 | 0.433245 |
|  | Talwar | 212.5487 | 7.478596 | 30.2438 | 1163.238 | 1163.473 | 1171.228 | 1174.228 | 0.427242 | 0.416121 |
|  | Welsch | 207.9983 | 6.588555 | 27.9739 | 1163.69 | 1163.925 | 1171.68 | 1174.68 | 0.435121 | 0.424153 |
| Chapman-Richards | Andrews | 1259.933 | 0.413938 | 0.00353 | 1208.315 | 1208.551 | 1216.306 | 1219.306 | 0.068572 | 0.059616 |
|  | Bisquare | 1256.642 | 0.413503 | 0.003529 | 1208.309 | 1208.545 | 1216.3 | 1219.3 | 0.068402 | 0.05944 |
|  | Cauchy | 1448.218 | 0.437156 | 0.003552 | 1208.831 | 1209.066 | 1216.821 | 1219.821 | 0.077712 | 0.068844 |
|  | Fair | **1647.803** | **0.479234** | **0.004703** | **1211.674** | **1211.909** | **1219.664** | **1222.664** | **0.102249** | **0.093617** |
|  | Huber | 1202.558 | 0.401416 | 0.003284 | 1208.007 | 1208.242 | 1215.998 | 1218.998 | 0.062625 | 0.053612 |
|  | Logistic | 1519.85 | 0.446033 | 0.00360 | 1209.137 | 1209.372 | 1217.127 | 1220.127 | 0.081685 | 0.072855 |
|  | Talwar | 820.5928 | 0.354522 | 0.004263 | 1207.869 | 1208.105 | 1215.86 | 1218.86 | 0.049474 | 0.040334 |
|  | Welsch | 1326.006 | 0.421539 | 0.003491 | 1208.444 | 1208.679 | 1216.434 | 1219.434 | 0.071383 | 0.062454 |
| Logistic | Andrews | 11.08621 | 1.107162 | 29.23067 | 606.0539 | 606.4499 | 616.7077 | 620.7077 | 0.279644 | 0.258457 |
|  | Bisquare | 11.08367 | 1.105991 | 29.21602 | 606.0699 | 606.466 | 616.7237 | 620.7237 | 0.27939 | 0.258196 |
|  | Cauchy | 11.08001 | 1.343946 | 31.42226 | 603.4931 | 603.8891 | 614.1469 | 618.1469 | 0.279012 | 0.257806 |
|  | Fair | **11.15196** | **1.557271** | **33.0482** | **601.2195** | **601.6155** | **611.8732** | **615.8732** | **0.297238** | **0.276568** |
|  | Huber | 11.1867 | 1.433542 | 31.65642 | 603.3735 | 603.7695 | 614.0272 | 618.0272 | 0.265824 | 0.24423 |
|  | Logistic | 11.12535 | 1.453336 | 32.21496 | 602.5171 | 602.9131 | 613.1708 | 617.1708 | 0.281027 | 0.25988 |
|  | Talwar | 11.72388 | 1.342784 | 30.91821 | 604.17 | 604.566 | 614.8237 | 618.8237 | 0.285863 | 0.264859 |
|  | Welsch | 11.06701 | 1.163746 | 29.82806 | 605.3329 | 605.729 | 615.9867 | 619.9867 | 0.278718 | 0.257504 |
| Weibull | Andrews | **7.393411** | **1.645111** | **-2.95725** | **643.7851** | **644.0204** | **651.7754** | **654.7754** | **0.085934** | **0.068185** |
|  | Bisquare | 7.39164 | 1.64627 | -2.95779 | 643.8261 | 644.0614 | 651.8164 | 654.8164 | 0.085805 | 0.068054 |
|  | Cauchy | 7.460944 | 1.602384 | -2.16795 | 640.4154 | 640.6507 | 648.4057 | 651.4057 | 0.065128 | 0.046976 |
|  | Fair | 7.584318 | 1.574767 | -1.72908 | 635.466 | 635.7012 | 643.4563 | 646.4563 | 0.058944 | 0.040671 |
|  | Huber | 7.439716 | 1.65232 | -1.89129 | 644.1934 | 644.4287 | 652.1837 | 655.1837 | 0.047974 | 0.029488 |
|  | Logistic | 7.501804 | 1.601948 | -1.86993 | 638.3054 | 638.5407 | 646.2957 | 649.2957 | 0.056068 | 0.037739 |
|  | Talwar | 7.635889 | 1.594309 | -3.09229 | 641.7516 | 641.9869 | 649.7419 | 652.7419 | 0.082303 | 0.064483 |
|  | Welsch | 7.400843 | 1.635663 | -2.87241 | 643.6321 | 643.8674 | 651.6224 | 654.6224 | 0.084373 | 0.066593 |
| Logistic | Andrews | 8.877001 | 3.348832 | 28.55586 | 453.5497 | 453.785 | 461.54 | 464.54 | 0.437852 | 0.426937 |
|  | Bisquare | 8.87678 | 3.348598 | 28.55336 | 453.5613 | 453.7966 | 461.5516 | 464.5516 | 0.437592 | 0.426672 |
|  | Cauchy | 8.87409 | 3.493757 | 29.36023 | 453.5168 | 453.7521 | 461.5071 | 464.5071 | 0.463009 | 0.452582 |
|  | Fair | **8.899556** | **3.662713** | **30.24308** | **453.6439** | **453.8792** | **461.6342** | **464.6342** | **0.509192** | **0.499661** |
|  | Huber | 8.872178 | 3.364235 | 28.57457 | 453.4407 | 453.676 | 461.431 | 464.431 | 0.428613 | 0.417519 |
|  | Logistic | 8.876045 | 3.531658 | 29.57957 | 453.4724 | 453.7077 | 461.4628 | 464.4628 | 0.4717 | 0.461442 |
|  | Talwar | 8.970344 | 2.643547 | 24.19661 | 452.2247 | 452.46 | 460.2151 | 463.2151 | 0.347543 | 0.334874 |
|  | Welsch | 8.87428 | 3.408435 | 28.88126 | 453.5861 | 453.8214 | 461.5764 | 464.5764 | 0.446925 | 0.436186 |
| Chapman-Richards | Andrews | 132.8098 | 0.546999 | 0.004358 | 475.8013 | 476.0366 | 483.7916 | 486.7916 | 0.28678 | 0.279922 |
|  | Bisquare | 132.818 | 0.546952 | 0.004355 | 475.7992 | 476.0345 | 483.7895 | 486.7895 | 0.28657 | 0.27971 |
|  | Cauchy | 130.69 | 0.555554 | 0.004917 | 476.3252 | 476.5605 | 484.3155 | 487.3155 | 0.30776 | 0.301104 |
|  | Fair | **127.7204** | **0.56794** | **0.005809** | **476.6953** | **476.9306** | **484.6856** | **487.6856** | **0.352485** | **0.346259** |
|  | Huber | 133.0662 | 0.545648 | 0.004258 | 475.036 | 475.2713 | 483.0263 | 486.0263 | 0.273775 | 0.266792 |
|  | Logistic | 129.7434 | 0.558088 | 0.005116 | 476.4705 | 476.7058 | 484.4608 | 487.4608 | 0.315582 | 0.309001 |
|  | Talwar | 118.581 | 0.487019 | 0.002641 | 474.3045 | 474.5398 | 482.2948 | 485.2948 | 0.196483 | 0.188757 |
|  | Welsch | 132.1662 | 0.550384 | 0.00456 | 476.0051 | 476.2404 | 483.9955 | 486.9955 | 0.294134 | 0.287347 |
